# Tailoring a sexual health curriculum to the sexual health challenges seen by midwifery, nursing and medical providers and students in Tanzania

**DOI:** 10.4102/phcfm.v14i1.3434

**Published:** 2022-05-31

**Authors:** B.R. Simon Rosser, Dickson A. Mkoka, Corissa T. Rohloff, Lucy R. Mgopa, Michael W. Ross, Gift G. Lukumay, Inari Mohammed, Agnes F. Massae, Ever Mkonyi, Stella E. Mushy, Dorkasi L. Mwakawanga, Nidhi Kohli, Maria E. Trent, James Wadley, Zobeida E. Bonilla

**Affiliations:** 1 Division of Epidemiology and Community Health, University of Minnesota, Minneapolis, United States of America; 2 Department of Clinical Nursing, Muhimbili University of Health and Allied Sciences, Dar es Salaam, United Republic of Tanzania; 3 Department of Educational Psychology, University of Minnesota, Minneapolis, United States of America; 4 Department of Psychiatric and Mental Health, School of Medicine, Muhimbili University of Health and Allied Sciences, Dar es Salaam, United Republic of Tanzania; 5 Department of Family Medicine and Community Health, University of Minnesota, Minneapolis, United States of America; 6 Department of Community Health Nursing, Muhimbili University of Health and Allied Sciences, Dar es Salaam, United Republic of Tanzania; 7 Department of Pediatrics and Adolescent Medicine, Johns Hopkins University, Baltimore, United States of America; 8 Department of Counseling and Human Services, Lincoln University, Philadelphia, United States of America

**Keywords:** sexual health, schools, medical, schools, nursing, curriculum, HIV infections, sexually transmitted diseases, sexual violence, sexual dysfunction, sexual and gender minorities, masturbation

## Abstract

**Background:**

Tanzania is a country experiencing multiple sexual health challenges, but providers receive no formal training in sexual health.

**Aim:**

This study aimed to assess (1) what sexual health challenges are commonly seen in clinics in Tanzania, (2) which are raised by patients, (3) which are not addressed and (4) which topics to prioritise for a sexual health curriculum.

**Setting:**

Healthcare settings in Tanzania.

**Methods:**

Participants were 60 experienced and 61 student doctors, nurses and midwives working in Dar es Salaam. The authors conducted 18 focus groups stratified by profession (midwifery, nursing or medicine) and experience (practitioners vs. students).

**Results:**

Providers identified six common sexual health concerns: (1) Human immunodeficiency virus (HIV) and acquired immunodeficiency syndrome (AIDS) and sexually transmissible infection (STI) (especially syphilis and gonorrhoea), (2) sexual violence (including intimate partner violence and female genital mutilation), (3) early and unwanted pregnancy (including early sexual debut and complications from abortion), (4) sexual dysfunctions, (5) key population concerns (e.g. lesbian, gay, bisexual, transgender (LGBT); sex work) and (6) non-procreative sexual behaviour (including pornography and masturbation in males and oral and anal sex practices in heterosexual couples). Across professions, few differences were observed. Homosexuality, sex work, masturbation and pornography were identified as taboo topics rarely discussed. Most participants (81%) wanted one comprehensive sexual health curriculum delivered across disciplines.

**Conclusion:**

A sexual health curriculum for health students in Tanzania needs to address the most common sexual health concerns of patients. In addition to teaching sexual science and clinical care, skills training in how to address taboo topics is recommended. Students endorsed almost all sexual health topics, which suggests that a comprehensive curriculum is appropriate.

## Introduction

### Lack of Sexual Health Training for Healthcare Providers

The concept of sexual health was initially articulated by the World Health Organization (WHO) in 1975.^1^ In high-income countries such as the United States (US), it is widely recognised as an ‘intrinsic element of human health’^2,3^ and integrated into the nation’s health goals.^4^ Despite this, only a minority of medical schools in the US have a formal sexual health curriculum integrated into their training,^5^ with between 42% and 62% of medical students reporting their training on sexuality and sexual health as inadequate.^6^ In low- and middle-income countries (LMICs), curricula to train health students in sexual health are even more rare. For example, with the exception of South Africa, there are no curricula for training future health professionals in sexual health across sub-Saharan Africa. The impact of this lack of patient care was highlighted in a recent study of 151 videotaped consultations with patients with hypertension and diabetes in the North West province, South Africa.^7^ Sexual histories were taken in only 3% of cases, patients living with sexual dysfunctions were missed, risk of human immunodeficiency virus (HIV) was not assessed and patients encountered provider paternalism and a lack of warmth and respect.^7^

The long-term objective of this study is to develop and evaluate a sexual health curriculum for healthcare students tailored for the sub-Saharan context. A core principle of public health interventions in LMICs is that they should be tailored to the country and its local epidemiology rather than simply imported from high-income countries.^8,9^ Understanding the sexual and reproductive health challenges of a country is the first essential step in identifying the challenges providers face, and prioritising content to ensure a sexual health curriculum is culturally relevant and responsive to the local conditions.^10^ To tailor a health curriculum to a country or region, two things are required. Firstly, an epidemiologic profile of the prevalence and incidence of sexual health concerns is required. Secondly, research needs to be conducted to identify the common patient concerns and presenting issues (such as that conducted in this analysis).

### Epidemiologic profile of the sexual health of Tanzania

As a country in sub-Saharan Africa, Tanzania faces multiple complex sexual and reproductive health challenges. Sub-Saharan Africa is the epicentre of the HIV pandemic. In Tanzania, 1.5 (95% confidence interval [CI]: 1.3–1.9) million or 5.3% of Tanzanians are estimated to be living with HIV. Of these, most (57.1%) are female.^11^ Human immunodeficiency virus prevalence amongst youth is high (at 2.0%).^11^ Youth also have low rates of accurate HIV knowledge (43.4%) and most (55.7%) report high-risk behaviour with a non-marital partner.^11^ Sub-Saharan Africa also has the highest rates of (non-HIV) sexually transmissible infections (STIs) in the world.^12^ In Tanzania, 2.7% of women are infertile (likely because of STIs),^13^ whilst 2.5% (95% CI: 2.3–3.6) of pregnant women are infected with syphilis.^14^

Women, sexual minorities and children and youth are three groups at higher risk of sexual health problems across multiple metrics. The sexual health of girls and women face multiple challenges. Until recently, 40.0% of girls were married off before the age of 18 years. In 2019, Tanzania raised the age of marriage to 18 years after human rights groups highlighted child marriage of girls as young as 9 years.^15^ In Tanzania, 54.1% of pregnancies were intended, 32.5% unintended and 13.4% unintended and unwanted.^16^ Early sexual debut and early marriage result in the world’s highest rates of teen pregnancy and poor pregnancy outcomes. In Dar es Salaam (study site), 27.0% of pregnant girls had severe wasting and almost half (48.0%) of babies had low birth weight.^17^ Abortion is illegal but appears to be a common practice. In 2008, East Africa had an estimated two million unsafe abortions (amongst 3.6% of women aged 15–44 years).^18^ Illegal abortion is estimated to cause 16.0% – 25.0% of maternal deaths.^19^ Most women who seek abortion are described as ‘young, single and desperate’.^20^

Girls and women also experience high rates of sexual violence. Intimate partner violence is reported by 33.0% of urban and 47.0% of rural women.^21^ Intimate partner violence is promoted and interpreted by some tribes as a sign of love.^21^ In rural areas, almost one in five (17.0%) girls describe their first sexual experience as forced.^22^ Female genital mutilation^23^ is now illegal but almost half (45.2%) of women have been circumcised.^24^

Tanzania is a conservative country where access to sex education is limited. Amongst adolescent boys, 17% interpret masturbation negatively.^25^ On an online forum for young Tanzanians, ‘discussion on the negative effects of masturbation … which types of sex are “normal” [*or*] “immoral” attracts a lot of attention’.^26^ Physical and sexual violence against children is common. Sexual violence is reported by 28% of girls and 13% of boys.^27^ Amongst adult women, 23% in urban areas and 31% in rural areas have been sexually assaulted.^21^ Sexual health education and access to care is also a concern for adults. For example, diabetes is an under-recognised health condition in this region^28^ and contributes to high rates of male erectile dysfunction.^29,30^

Homosexuality is both a criminal offense and heavily stigmatised. The punishment for sodomy is up to life imprisonment for men and, in Zanzibar, up to five years for women. In 2016, Justice Minister Mwakyembe announced that groups advocating for LGBT rights were illegal, proposed arresting anyone on Facebook with pro-gay sentiments and banned sexual lubricant (on the rationale that it facilitated homosexual sex).^31^ Men who have sex with men (MSM) are reluctant to seek HIV and STI testing and care out of fear of being shunned or arrested. As a consequence, they have high rates of undiagnosed HIV and STIs.^32,33,34^ Almost a third (29.5%) of MSM report being physically attacked for being MSM.^32^ Cases of ‘corrective rape’ of lesbians have also been reported.^35^

Healthcare workers in Tanzania have had little to no access to training in sexual healthcare. Whilst this gap in training impacts all patient groups, high-risk populations may be most affected. Indeed, high-risk groups identify the attitudes of healthcare workers as a major barrier to sexual and reproductive health care.^36^

Firstly, our long-term goal in this study was to develop and evaluate a comprehensive sexual health curriculum for health students in sub-Saharan Africa universities. For a training curriculum to be relevant to and maximally effective in Africa, it needs to be African-centric, that is, grounded in both the epidemiology (as described here) and the real clinical problems seen by providers. Whilst the given statistics attest to a nation with major sexual health challenges, this study was conducted firstly to find out what sexual health challenges are commonly seen by healthcare providers in Tanzania, which are raised by patients and which may be suspected but not currently addressed in the clinical context. Secondly, the study was conducted to identify whether the three main disciplines providing clinical care in Tanzania (midwives, nurses and medical doctors) saw similar types of sexual health concerns or whether each discipline specialised in the type of sexual health problems treated. (It should be noticed that because of a shortage of clinicians in Tanzania, many midwives and nurses may treat a wider range of patients than their peers in other countries). Thirdly, a needs assessment was conducted to identify potential topics for a sexual health curriculum and learn whether a common curriculum across professions or one tailored to each specialty was preferred.

## Methodology

### Study design

This formative research is part of a larger mixed methods research study developing and evaluating an Afrocentric sexual health training programme for midwifery, nursing and medical students attending Muhimbili University of Health and Allied Sciences (MUHAS) in Dar es Salaam, Tanzania. The methods for this study have been published elsewhere.^37^ To summarise, we utilised a stratified three (profession: midwifery, nursing, medicine) by two (experience: clinicians vs students) design. The main objective was to assess and compare how providers address sexual health concerns across professions and by experience (providers vs students) in order to identify curriculum priorities. Focus groups were chosen because they are an efficient method to obtain information about clinical practices in a resource-limited and conservative environment.

### Study participants

Study participants included healthcare students and licensed providers. The healthcare providers were nurses, midwives and medical doctors with at least two years’ clinical experience, recruited mainly from three public and private hospitals. These were Muhimbili National Hospital, Mnazi Mmoja Hospital and Agakhan Hospital, all located at Ilala District, Dar es Salaam. Purposive sampling methods were employed to recruit a diverse sample of 61 experienced providers.^38^ If there was more than one provider who met eligibility criteria, the provider with the most experience and expertise was enrolled.

Sixty-one students were enrolled from midwifery, nursing and medicine in their fourth year (final year for nurses and midwives and penultimate year for medical students). Fliers and announcements were placed around campus halls at MUHAS, and interested students were requested to contact the study staff at MUHAS.

### Study procedures

In June 2019, a total of 18 focus group discussions were conducted in a period of one month, using a stratified, cross-sectional study design. Groups were stratified using a two experience (student or experienced health provider) by three discipline (midwifery, nursing, medicine) design with three focus groups per cell to achieve saturation. This stratification was to allow us to observe differences across professions and clinical experiences.

Each group had between five to eight participants and lasted 60 to 90 min. To ensure confidentiality, the focus groups were held in convenient private rooms located within the hospital premises or at the university. The focus group moderator welcomed the participants and offered them refreshments to make them feel comfortable. Participants were provided with pen and paper to write their brief demographic characteristics (i.e. age, years of experience or clinical rotation, department) and a table card to write their first name or a nick name. Each group was led by a bilingual moderator assisted by a co-moderator who took notes and observed non-verbal expressions of the participants. Whilst participants were told that they could discuss in English or Kiswahili, whichever they preferred, the questions were posed in Kiswahili and the discussion was mostly in Kiswahili. The following set of ground rules was shared in every focus group: to inform participants about their voluntary participation, rights to withdraw from the interview at any time, to digitally audio-record the group and the limits of confidentiality. Participants received 60 000 Tanzanian Shillings (about $25.00) to cover travel and other costs that they may have incurred.

The discussion was based on a semi-structured interview that started with an opening question about their current practice (for providers) or most recent clinical rotation (for students). Then, participants were asked four questions to identify the most common sexual health challenges in Tanzania.

The moderator started by announcing that the topic of this focus group was ‘the sexual health of patients in Tanzania and the type of sexual problems that patients have’. The moderator then defined ‘sexual problems’ as ‘everything related to sex, broadly speaking’. Then, the moderator handed out cards and introduced the first exercise.

The first exercise asked participants to ‘write down the five most common sexual health problems you think your patients have (whether or not they talk about them with you)’. These were reported on a white board with the follow-up prompt: ‘Are there other important problems that are common in Tanzania that you don’t see in your particular practice?’ The second question asked participants to place a check mark beside the two most common problems patients talk about with their provider. Then, a follow-up prompt asked them to discuss how these problems differ by patient characteristics (e.g. gender, income, level of education or rural or urban location or tribal affiliation).

These questions were followed by a series of clinical case studies in which members of the groups were asked to give their opinions on how each case would get treated or not treated in Tanzania. Case presentations included male erectile dysfunction, intimate partner violence, female dyspareunia and vaginal warts, a 14-year-old girl requesting contraception, sexual abuse of a 9-year-old male, male homosexual behaviour, a rape victim and finally, a case involving adolescent masturbation. Following these, participants completed a pen-and-paper survey to identify potential topics for a sexual health training, what they had already received training in, the formats for instruction and comments on such a curriculum. The final question asked if participants would recommend students to learn using a common curriculum versus a curriculum tailored for each discipline. The question was phrased as follows: Finally, we would like your advice. Some members of the team think our midwifery, nursing and medical students should all get the same module so they can learn together, while others think each group should have a separate module. What do you think?

This analysis reports the results of the questions on what the most common sexual health problems are that patients have in Tanzania and which are raised by patients and on whether a common curriculum versus tailored curricula was preferred.

### Data management and analysis

At the end of each session, the cards of each participant were collected by the moderator. Quantitative data, including the participant demographic characteristics and the answers on the cards, were entered into an Excel spreadsheet and analysed. Once all responses were logged, the responses were grouped into logical groups. If a comment mentioned multiple categories (e.g. ‘STIs including syphilis and gonorrhoea’) it was entered in each category (i.e. ‘STIs’, ‘syphilis’ and ‘gonorrhoea’ all scored a point). (Note: this only affected a few [< 5%] of the responses.) In this mixed-methods analysis, the frequency of each category by profession were counted. These were then expressed as an overall percentage. For the fourth question, transcripts were coded using inductive and deductive methods, as informed by grounded theory principles.

Pearson’s chi-square test for count data was used to compare the proportion of comments generated for each topic by profession (midwifery, nursing, medicine) and separately by experience (student, licensed provider). Sexual health topics with an expected frequency less than five were removed from these analyses. When the chi-square was significant (at *p* < 0.05), pairwise comparisons with a Benjamin–Hochberg correction for multiple comparisons were used to identify where the significant differences occurred.

### Ethical considerations

This study was a collaboration between researchers at the University of Minnesota, United States of America and the MUHAS, Tanzania. The Institutional Review Boards of the (Tanzania) National Institute of Medical Research (NIMR) approved the study at MUHAS (NIMR/HQ/R.8c.Vol.I/897) as did the University of Minnesota (number: STUDY00010341). Ethical clearance exempted the study from further human subjects review since the focus was on clinical practice and development of the curriculum (IRB STUDY00006904). The study was also registered as a clinical trial (NCT03923582) because its aim included a randomised controlled trial of the Afrocentric sexual health curriculum.

## Results

As detailed in Table 1^37^, there were 60 experienced healthcare providers, including 11 men and 49 women. Their ages ranged from 24 to 62 years, and had, on average, 15 years of clinical experience. Of the 61 students, 38 were men and 23 were women. Their age distribution ranged between 22 years and 37 years, and all students were in their fourth year of their clinical programmes.

**TABLE 1:** Demographic characteristics of the focus group participants.

Demographic	Midwifery	Nursing	Medicine	Total
**Sample size**				
Students	20	19	22	61
Providers	21	21	18	60
Total	41	40	40	121
**Gender**				
Students				
Male	14	13	11	38
Female	6	6	11	23
Providers				
Male	0	5	6	11
Female	21	16	12	49
Total				
Male	14	18	17	49
Female	27	22	23	72
**Age (in years)**				
Students				
Mean	27.7	25.1	24.0	25.5
Range	23–37	23–27	22–28	23–37
Providers				
Mean	44.5	41.1	43.5	43.1
Range	26–58	24–59	31–62	24–62
**Experience (in years)**				
Students	4	4	4	4
Providers				
Mean	18.0	13.1	11.9	14.6
Range	4–38	2–30	4–25	2–38

*Source:* Mgopa LR, Rosser BS, Ross MW, et al. Cultural and clinical challenges in sexual health care provision to men who have sex with men in Tanzania: A qualitative study of health professionals’ experiences and health students’ perspectives. BMC Public Health. 2021;21(1):1–12. https://doi.org/10.1186/s12889-021-10696-x

Tables 2 and 3 detail the most common sexual health problems of patients in Tanzania. In order of the most frequently cited problems, participants cited HIV and STIs, sexual violence, early or unwanted pregnancy, sexual dysfunction and concerns about homosexuality and non-procreative sexual behaviour as common. In contrast, cultural and social sexual health concerns (e.g, polygamy, sex work) and family planning were rarely cited. The midwives and nurses were significantly more likely to cite sexual violence than the medical doctors (*p* < 0.05), and conversely, the medical doctors were more likely to cite male sexual dysfunction as a common concern than either the nurses (*p* < 0.05) or midwives (*p* < 0.001; see Table 2). The students and experienced professionals cited similar problems, although the students were possibly more likely to identify sexual violence and non-procreative sexual behaviours as common sexual health concerns than the experienced health professionals (*p* = 0.05; see Table 3).

**TABLE 2:** Most common sexual health problems patients have by profession.

Topic	Total	Midwifery		Nursing		Medicine		*χ* ^2^	*p*
		*n*	%	*n*	%	*n*	%		
HIV and STIs†	157	50	31.8	54	34.4	53	33.8	0.166	0.921
**Sexual violence‡**	**150**	**62**	**41.3^a^**	**54**	**36.0^b^**	**34**	**22.7^a,b^**	**8.32**	**0.016**
Early or unwanted pregnancy§	118	48	40.7	35	29.7	35	29.7	2.86	0.239
**Sexual dysfunction¶**	**75**	**12**	**16.0^c^**	**21**	**28.0^d^**	**42**	**56.0^c,d^**	**18.96**	**0.000**
LGBT and key populations††	33	13	39.4	11	33.3	9	27.3	0.727	0.695
Non-procreative sexual behaviours‡‡	26	8	30.8	13	50.0	5	19.2	3.77	0.152
Family planning§§	6	3	50.0	2	33.3	1	16.7	NA	NA
Societal issues¶¶	22	6	27.3	12	54.5	4	18.2	4.73	0.094
Other problems†††	10	4	40.0	0	0.0	6	60.0	NA	NA

Note: Responses based on question: ‘Question: Write down the five most common sexual health problems you think your patients have (whether or not they talk about them with you)’. Data in bold (superscripts a, b, c, d) denote the only significant differences. For example, on sexual violence, Midwifery (41.3%^a^) and Medicine (22.7%^a^) and Nursing (36.0%^b^) and Medicine (22.7%^b^) differed significantly while the difference between Midwifery (41.3%) and Nursing (36.0%) did not reach statistical significance.

HIV, human immunodeficiency virus; STI, sexually transmissible infections; LGBT, lesbian, gay, bisexual, transgender; NA, not applicable.

† , HIV and STIs were sometimes mentioned together or separately, with gonorrhoea and syphilis being the two non-HIV STIs most commonly emphasised;

‡ , Sexual violence included terms for rape, gender-based violence (GBV), intimate partner violence, as well as female genital mutilation (FGM);

§ , Early pregnancy included terms such as early pregnancy, unwanted pregnancy, teen pregnancy and the sequelae effects of abortion;

¶ , Sexual dysfunction was the most common term, sometimes clarified as male erectile disorder;

†† , LGBT was used about as frequently as homosexuality and some also referenced sex workers;

‡‡ , Non-procreative behaviours included especially masturbation and pornography (specific to males) and anal and oral sex practices (in heterosexual couples);

§§ , Family planning and contraceptives were the terms commonly used;

¶¶ , Societal issues included concerns about early sex, premarital and extramarital sex, kidnapping and promiscuity;

††† , Other problems included sexual challenges secondary to cancer or diabetes, menstruation difficulties and genital odour.

**TABLE 3:** Differences between students and providers in the sexual health concerns of patients.

Topic	Total	Student Provider		Provider		*χ* ^2^	*p*
	*n*	%	*n*	%			
HIV and STIs	157	86	54.8	71	45.2	1.43	0.231
**Sexual violence**	**150**	**87**	**58.0**	**63**	**42.0**	**3.84**	**0.050**
Early or unwanted pregnancy	118	60	50.8	58	49.2	0.034	0.854
Sexual dysfunction	75	41	54.7	34	45.3	0.653	0.419
LGBT and key populations	33	13	39.4	20	60.6	1.49	0.223
**Non-procreative sexual behaviours**	**26**	**18**	**69.2**	**8**	**30.8**	**3.85**	**0.050**
Family planning	6	1	16.7	5	83.3	NA	NA
Societal issues	22	7	31.8	15	68.2	2.91	0.088
Other problems	10	4	40.0	6	60.0	NA	NA

Note: Responses based on question: ‘Question: Write down the five most common sexual health problems you think your patients have (whether or not they talk about them with you)’**. Data in bold denote statistically significant differences.**

HIV, human immunodeficiency virus; STI, sexually transmissible infections; LGBT, lesbian, gay, bisexual, transgender; NA, not applicable.

Tables 4 and 5 show the common sexual health concerns that patients raise with their providers. Human immunodeficiency virus, STIs, sexual violence and early or unwanted pregnancy were common presenting concerns across providers, whilst sexual dysfunction was commonly reported by nurses and medical providers as well. In contrast, almost no one reported talking with patients about LGBT concerns and non-procreative behaviour. As seen in Table 4, nurses were more likely to report talking to patients about HIV and STIs than medical doctors (*p < 0.*05), medical doctors were significantly more likely to report patients raising sexual dysfunction concerns than midwives (*p* = 0.001) and midwives were more likely to report talking about sexual violence with patients than doctors (*p* < 0.05). In addition, students were significantly more likely to talk with patients about sexual violence than experienced providers (*p* < 0.05; see Table 5).

**TABLE 4:** Sexual health topics raised by patients.

Topic	Total	Midwifery		Nursing		Medicine		*χ* ^2^	*p*
		*n*	%	*n*	%	*n*	%		
**HIV and STIs**	**71**	**19**	**26.8**	**34**	**47.9**	**18**	**25.4**	**6.79**	**0.034**
**Sexual violence**	**49**	**22**	**44.9**	**19**	**38.8**	**8**	**16.3**	**6.65**	**0.036**
Early or unwanted pregnancy	40	11	27.5	14	35.0	15	37.5	0.650	0.723
Sexual dysfunction	29	2	6.9	9	31.0	18	62.1	13.31	0.001
LGBT and key populations	1	0	0.0	0	0.0	1	100.0	NA	NA
Non-procreative sexual behaviours	2	0	0.0	1	50.0	1	50.0	NA	NA
Family planning	1	0	0.0	0	0.0	1	100.0	NA	NA
Societal issues	7	2	28.6	3	42.9	2	28.6	NA	NA
Other problems	6	1	16.7	0	0.0	5	83.3	NA	NA

Note: Responses based on question: ‘Question: Now, write down the two most common ones they (your patients) talk about with you.’ Data in bold denote statistically significant differences.

HIV, human immunodeficiency virus; STI, sexually transmissible infections; LGBT, lesbian, gay, bisexual, transgender; NA, not applicable.

**TABLE 5:** Differences between students and providers in the sexual health topics raised by patients.

Topic	Total	Student		Provider		*χ* ^2^	*p*
		*n*	%	*n*	%		
HIV and STIs	71	40	56.3	31	43.7	1.14	0.285
**Sexual violence**	**49**	**33**	**67.3**	**16**	**32.7**	**5.90**	**0.015**
Early or unwanted pregnancy	40	21	52.5	19	47.5	0.100	0.752
Sexual dysfunction	29	12	41.4	17	58.6	0.862	0.353
LGBT and key populations	1	0	0.0	1	100.0	NA	NA
Non-procreative sexual behaviours	2	1	50.0	1	50.0	NA	NA
Family planning	1	0	0.0	1	100.0	NA	NA
Societal issues	7	2	28.6	5	71.4	NA	NA
Other problems	6	3	50.0	3	50.0	NA	NA

Note: Responses based on question: ‘Question: Now, write down the two most common ones they (your patients) talk about with you.’ Data in bold denote statistically significant differences.

HIV, human immunodeficiency virus; STI, sexually transmissible infections; LGBT, lesbian, gay, bisexual, transgender; NA, not applicable.

Most participants (81%) stated that they would prefer all students to receive the same curriculum together, rather than midwifery, nursing and medical students receiving tailored curricula for each profession (see Table 6). Amongst current students, 86.4% supported a common curriculum and 75.4% of experienced providers did as well. When asked what topics should be covered in a sexual health curriculum, most students endorsed all the topics as important (see Table 7). However, six topics were endorsed by more than 10.0% of participants as less necessary because they felt they had been trained in them already: STI infections (20.0%, including 38.0% from medicine and 10.0% from nursing and midwifery), HIV prevention (17.0%, including 28.0% from medicine, 15.0% from midwifery and 8.0% from nursing), HIV testing (15.0%, including 23.0% from medicine and 10.0% from midwifery and nursing) and male sexual dysfunction (14.0%, including 20.0% from medicine, 8.0% from midwifery and 3.0% from nursing). In each case, participants from medicine were more likely to state they had been trained in these than midwifery or nursing participants. Most preferred methods of instruction were formal lectures (*n* = 25), group discussion (*n* = 23), practice with real patients (*n* = 19), readings and research (*n* = 15), role plays (*n* = 14) and panels of patients (*n* = 11). Case studies (*n* = 7) and problem-based learning (*n* = 7) were the least preferred options.

**TABLE 6:** Preferred formats for sexual health education.

Format	Total		Midwifery		Nursing		Medical		*χ* ^2^	*p*	Students		Professional		*χ* ^2^	*p*
	*n*	%	*n*	%	*n*	%	*n*	%			*n*	%	*n*	%		
One curriculum	94	81.0	34	80.9	33	89.1	27	73.0	0.915	0.633	51	86.4	43	75.4	0.681	0.409
Tailored	22	19.0	8	19.0	4	10.8	10	27.0	2.55	0.280	8	13.6	14	24.6	1.64	0.201

Note: Responses based on question: The authors would like your advice. Some members of the team think our midwifery, nursing and medical students should all get the same module so they can learn together, whilst others think each group should have separate module. What do you think? Data in bold denote statistically significant differences.

**TABLE 7:** Potential topics to be covered in a sexual health curriculum for midwifery, nursing and medical students.

Topic	Total	Yes†		Unsure		No‡		No§	
		*n*	%	*n*	%	*n*	%	*n*	%
1 HIV testing	117	97	82.91	3	2.56	17	14.53	0	0.00
2 HIV prevention (e.g. how to use a condom)	116	94	81.03	2	1.72	20	17.24	0	0.00
3 What is normal sexual development across the lifespan?	115	95	82.61	10	8.70	10	8.70	0	0.00
4 How to take a sexual history	116	109	93.97	0	0.00	7	6.03	0	0.00
5 How to talk to/counsel patients about sexual health issues	117	110	94.02	3	2.56	3	2.56	1	0.85
6 How to treat difficulties getting pregnant	114	96	84.21	9	7.89	8	7.02	1	0.88
7 How to treat sexual assault and violence (e.g. rape)	116	107	92.24	6	5.17	2	1.72	1	0.86
8 Sex during pregnancy and after childbirth	116	94	81.03	10	8.62	11	9.48	1	0.86
9 How to talk with patients about a miscarriage	115	96	83.48	6	5.22	11	9.57	2	1.74
10 How to talk to children and young people about sex	117	106	90.60	4	3.42	6	5.13	1	0.85
11 How to write policies for Tanzania related to sexual health	114	90	78.95	18	15.79	3	2.63	3	2.63
12 How to address concerns about masturbation	116	100	86.21	10	8.62	2	1.72	4	3.45
13 How to treat sexual concerns of patients different from me (e.g. cultural and religious differences)	117	102	87.18	10	8.55	3	2.56	2	1.71
14 How to treat lesbian, gay, and bisexual patients	117	95	81.20	15	12.82	2	1.71	5	4.27
15 How to treat transgender patients	115	86	74.78	21	18.26	4	3.48	4	3.48
16 How to treat sex workers (e.g. HIV, violence prevention)	115	97	84.35	6	5.22	8	6.96	4	3.48
17 Sexual transmitted infections (e.g. syphilis, chlamydia)	116	90	77.59	3	2.59	23	19.83	0	0.00
18 Male sexual dysfunction (causes, counselling, treatments)	117	101	86.32	3	2.56	12	10.26	1	0.85
19 Female sexual dysfunction (causes, counselling, treatments)	117	107	91.45	2	1.71	7	5.98	1	0.85
20. Other please specify¶									

HIV, human immunodeficiency virus; PID, pelvic inflammatory disease; CaCx, cancer of the cervix; GBV, gender-based violence; AB, abortion.

† , Yes, this should be in the training;

‡ , No, I have already been well trained in this;

§ , No, I do not need to learn about this. This is unimportant;

¶ , Other responses included (one each); Sexual disorders, paraphilia, disorders of sexual differentiation, early pregnancy, Hepatitis B, family planning (teen), unsafe abortion, children’s upbringing related to sexual issues and contraceptives.

## Discussion

There are four key findings in this study which the authors wish to highlight. Firstly, participants identified the six to seven most common sexual health concerns that patients present with in Tanzania. Secondly, across professions and between students and licensed providers, few differences were observed, although midwives and nurses (who may be more likely to treat women) were more likely to identify sexual violence whilst doctors (who may be more likely to see men) emphasised male sexual dysfunction. Thirdly, there was a clear distinction between those concerns that patients presented with and were commonly discussed (i.e. HIV and STIs, sexual violence, pregnancy and sexual dysfunction) and concerns that providers saw as common, but which were almost never discussed (i.e. homosexuality, sex work and non-procreative behaviours including masturbation, pornography and oral and anal sex in heterosexual relationships). Fourthly, most participants endorsed almost all topics and most preferred a common curriculum across specialties. This suggests that a comprehensive sexual health curriculum may work best in health universities such as the one we studied in Tanzania.

The primary purpose of this research was to inform the development of an African-centric sexual health curriculum for healthcare students. The results suggest that to equip students to discuss and treat the common sexual health concerns seen in Tanzania, a sexual health curriculum needs to emphasise the following topics. They are HIV, AIDS and STIs (especially syphilis and gonorrhoea); sexual violence (especially intimate partner violence, rape and female genital mutilation); early and unwanted pregnancy (including early sexual debut and complications from abortion); sexual dysfunctions (especially erectile disorders); LGBT and key population concerns (especially homosexuality and sex work); and non-procreative sexual behaviour (including pornography use and masturbation in males and oral and anal sex practices in heterosexual couples). There appears a clear need also to train providers in how to address topics that are ‘taboo’ in Tanzania that they suspect patients have but which otherwise go unaddressed. These include training in how to address the healthcare of LGBT persons, sex workers and other key populations and education and skills in addressing concerns about homosexuality, masturbation, pornography use, anal and oral sex.

Across the focus groups, midwifery, nursing and medical providers and students all identified essentially the same topics and when asked to choose preferred topics, participants endorsed almost all items. Given this overlap, it can be interpreted to mean that a common comprehensive sexual health curriculum could be used across the three disciplines and this approach had wide support amongst the participants in this study. Given some specialisation, medical students may need extra training in the treatment of sexual dysfunction and increased recognition of sexual violence as a common concern in their patients. Conversely, midwifery and nursing students may need extra training in treating the effects of sexual violence and recognition of sexual dysfunction as a common patient concern.

Healthcare delivery is deeply rooted in the social and cultural context of a country. Tanzania is demographically and culturally diverse. An African-centric sexual health curriculum needs to reflect and respect this complexity, whilst educating students in the sexual science and clinical treatment of such concerns.

This study provides support against simply importing a sexual health curriculum from another country (and especially from high income countries in Europe or North America. Some behaviours viewed as deeply problematic or pathological in high income countries (e.g. intimate partner violence and female genital mutilation) were defended by some participants as culturally appropriate (or even necessary in some Tanzanian sub-cultures). Conversely, behaviour viewed as non-pathological and legal in high income countries (e.g. homosexuality, non-procreative sexual behaviour in heterosexual persons and use of lubricant) is deeply stigmatised and illegal in Tanzania. Stigma may create anxieties in both patient and provider, so providers need training in the science supporting or refuting an association between sexual expression and pathology.

Consistent with Tanzania being a conservative country where sexual health concerns are not discussed, the authors highlight that the highest endorsed items in the list of potential topics were as follows: how to talk to counsel patients about sexual health issues (94%), how to take a sexual history (94%), how to treat sexual assault and violence (92%), how to talk to children and young people about sex (91%) and female sexual dysfunction (91%). The authors recommend that sexual health training include a strong skills building component including opportunities to practice history taking and counselling skills. One difference in presenting concerns between Tanzania and Western countries was the lack of endorsement of family planning as a common presenting clinical concern in Tanzania. Other sexual health issues may simply have a different context. For example, marriage is a little more complex in Tanzania where both monogamous marriages for Christians and monogamous and polygamous marriages for Muslim and other groups are recognised as legal. And compared with the West, Tanzanians place a greater emphasis on family, community and tribe. This makes clinical advice about what sexual behaviour is acceptable and more multilayered than in Western countries. Tanzania experiences several sexual health challenges common to other low-income countries with reliance on tourism. These include sex work, sugar daddies, survival sex, the sexual exploitation of children, differing standards of dress, behaviour and accountability for locals versus tourists and limited sexual healthcare resources for treatment and care. The clinical care of patients in these circumstances also needs to be addressed in an African-centric sexual health curriculum.

There are several limitations to consider in interpreting these findings. Firstly, the research reported in this article is only one part of a larger study. Secondly, these are health student and provider perceptions informed by their clinical experience and knowledge of studies conducted in Tanzania. It is possible that some perceptions may be faulty, either because a sexual health concern has been exaggerated (e.g. when an issue becomes politicised) or minimised (e.g. many sexual issues are hidden). Thirdly, we were not able to link the participants’ demographics to the comments. It is possible that some of the differences observed between professionals (e.g. between midwifery and medicine) may actually be accounted for by gender or other differences in the providers or in their patients and not in the profession itself. Fourthly, the unit of measurement was providers being asked to write down their patients’ most common presenting concerns. As some providers wrote meta-categories (e.g. HIV and STIs) whilst others wrote in finer detail (e.g. gonorrhoea, syphilis), the number of responses was not even across participants.

### Future directions

The next stage in our research is to adapt a comprehensive sexual health curriculum informed by these and other research findings in order to make it African-centric and as relevant and clinically useful as possible. Then we will conduct a randomised controlled trial to evaluate the effects of the curriculum on healthcare students’ knowledge, attitudes and skills in addressing sexual health concerns.

There are several directions for future research. Within Tanzania, translational studies are needed to address how best to train the healthcare workforce to be able to address the sexual health concerns of patients in Tanzania. In addition to training students, continuing education and perhaps online training may help disseminate the curriculum to providers. Studies in other countries across sub-Saharan Africa will test the generalisability of these findings and need for further tailoring by country. Should the sexual health curriculum prove effective, its generalisability should be tested at other health universities across the continent.

## Conclusion

This analysis reports the results of a formative research study to identify the six to seven most common sexual health concerns that patients present with in Tanzania. Participants made a clear distinction between those concerns that patients presented with which were commonly discussed (i.e. HIV and STIs, sexual violence, pregnancy and sexual dysfunction) and concerns that providers saw as common, but which were almost never discussed (i.e. homosexuality, sex work and non-procreative behaviours including masturbation, pornography and oral and anal sex in heterosexual relationships). Across professions and between students and licensed providers, few differences were observed. This suggests that a comprehensive sexual health curriculum may work best in health universities such as the one studied in Tanzania. A curriculum based on these findings has been developed for midwifery, nursing and medical students and is currently being rigorously evaluated in a randomised controlled trial.
